# TGR5 activation attenuates neuroinflammation via Pellino3 inhibition of caspase-8/NLRP3 after middle cerebral artery occlusion in rats

**DOI:** 10.1186/s12974-021-02087-1

**Published:** 2021-02-02

**Authors:** Hui Liang, Nathanael Matei, Devin W. McBride, Yang Xu, Zhenhua Zhou, Jiping Tang, Benyan Luo, John H. Zhang

**Affiliations:** 1 Department of Neurology, The First Affiliated Hospital, Zhejiang University School of Medicine, Hangzhou, China; 2 Department of Physiology and Pharmacology and Department of Anesthesiology, Loma Linda University, 11041 Campus St, Risley Hall, Room 219, Loma Linda, CA 92354 USA; 3 The Vivian L. Smith Department of Neurosurgery, McGovern Medical School, The University of Texas Health Science Center at Houston, Houston, TX USA

**Keywords:** TGR5, Inflammation, Neuroprotection, Middle cerebral artery occlusion, Early brain injury

## Abstract

**Background:**

Nucleotide-binding oligomerization domain-like receptor pyrin domain-containing protein 3 (NLRP3) plays an important role in mediating inflammatory responses during ischemic stroke. Bile acid receptor Takeda-G-protein-receptor-5 (TGR5) has been identified as an important component in regulating brain inflammatory responses. In this study, we investigated the mechanism of TGR5 in alleviating neuroinflammation after middle cerebral artery occlusion (MCAO).

**Methods:**

Sprague-Dawley rats were subjected to MCAO and TGR5 agonist INT777 was administered intranasally 1 h after MCAO. Small interfering RNAs (siRNA) targeting TGR5 and Pellino3 were administered through intracerebroventricular injection 48 h before MCAO. Infarct volumes and neurologic scores were evaluated, and ELISA, flow cytometry, immunofluorescence staining, immunoblotting, and co-immunoprecipitation were used for the evaluations.

**Results:**

Endogenous TGR5 and Pellino3 levels increased after MCAO. TGR5 activation by INT777 significantly decreased pro-inflammatory cytokine, cleaved caspase-8, and NLRP3 levels, thereby reducing brain infarctions; both short- and long-term neurobehavioral assessments showed improvements. Ischemic damage induced the interaction of TGR5 with Pellino3. Knockdown of either TGR5 or Pellino3 increased the accumulation of cleaved caspase-8 and NLRP3, aggravated cerebral impairments, and abolished the anti-inflammatory effects of INT777 after MCAO.

**Conclusions:**

TGR5 activation attenuated brain injury by inhibiting neuroinflammation after MCAO, which could be mediated by Pellino3 inhibition of caspase-8/NLRP3.

**Supplementary Information:**

The online version contains supplementary material available at 10.1186/s12974-021-02087-1.

## Background

Stroke is a leading cause of death and disability worldwide, affecting millions of lives every year [1]. Accumulating evidence suggests that innate immunity and inflammatory responses are involved in ischemic brain injury [2, 3]. Recent findings demonstrate that the nucleotide-binding oligomerization domain-like receptor pyrin domain-containing protein 3 (NLRP3) inflammasome, which is abundantly expressed in the brain, plays an important role in detecting cellular damage and mediating inflammatory responses to aseptic tissue injury during ischemic stroke [4–6]. Research has also shown that pharmacologic targeting of the NLRP3-mediated inflammatory response could help in the development of therapeutic strategies to prevent the deterioration of cerebral function [4].

Trans-membrane G protein-coupled receptor-5 (TGR5) is a plasma membrane-bound G protein-coupled bile acid receptor; this protein has varied expression levels in different tissues [7, 8]. Previous studies have shown that activation of TGR5 suppresses proinflammatory cytokine production and phagocytosis by monocytes/macrophages [7, 9]. Recent research has demonstrated that TGR5 activation blocks NLRP3 inflammasome-dependent inflammation, including lipopolysaccharide (LPS)-induced systemic inflammation, alum-induced peritoneal inflammation, and type-2 diabetes-related inflammation [10]. In central nervous system (CNS) research, studies have shown that TGR5 activation alleviates neuroinflammation and improves outcomes in models of experimental autoimmune encephalomyelitis and hepatic encephalopathy [11, 12]. Our previous research showed that TGR5 activation alleviates brain injury following middle cerebral artery occlusion (MCAO) [13]. However, the effects of TGR5 on neuroinflammation after ischemic stroke have not been investigated.

Pellino3 is an E3 ubiquitin ligase protein with anti-inflammatory properties [14, 15]. Pellino3 reduces caspase-8 cleavage and inhibits tumor necrosis factor-α (TNFα)-induced cell death [16]. Several papers have reported that caspase-8 activates NLRP3 in human monocytes, intraocular pressure-induced retinal ischemia, and after chemotherapeutic treatment [17–19]. Recent research indicates that caspase-8 inhibition decreases the neuropathological consequences of cerebral or retinal infarction and that TGR5 inhibits caspase-8 activation after liver ischemia/reperfusion injury [20, 21].

In the present study, we hypothesized that (1) TGR5 activation attenuates neuroinflammation after MCAO and (2) the anti-inflammatory mechanism of TGR5 activation is mediated by Pellino3 inhibition of caspase-8/NLRP3.

## Materials and methods

All protocols were approved by the Institutional Animal Care and Use Committees of Loma Linda University and Zhejiang University. All animal care and use were conducted in accordance with the Guide for the Care and Use of Laboratory Animals of the National Research Council. All experiments are reported in compliance with the ARRIVE (Animal Research: Reporting in Vivo Experiments) guidelines (http://www.nc3rs.org.uk/arrive). A total of 397 Sprague-Dawley male rats (2–3 months, weight 250–300 g) were used (Supplemental Table 1), and the investigators were blinded to group assignments during outcome assessments.

### MCAO model

Transient MCAO was induced as described previously [22], with some modifications. Briefly, anesthesia was induced by intraperitoneal administration with ketamine (80 mg/kg) and xylazine (10 mg/kg). Atropine (0.1 mg/kg, subcutaneous) was then administered. The right common carotid artery (CCA), internal carotid artery (ICA), and external carotid artery (ECA) were exposed surgically. The ECA was ligated, and a 4-0 nylon suture with silicon was inserted into the ICA through the ECA stump to occlude the MCA. The suture was removed after 2 h of occlusion. Sham-operated rats underwent the same surgical procedures except that the MCA was not occluded. After closing the skin incision, rats were maintained at approximately 37 °C on an electric heating blanket until the animals completely recovered from anesthetic.

### Experimental design

The experimental design is shown in Supplemental figure 1.

#### Experiment 1

For immunoblot determination of the time course of endogenous TGR5 and Pellino3 levels after MCAO, 30 rats were divided into 5 groups of 6 rats each, including: Sham, and at each of 4 different time points (6, 12, 24, and 72 h) after completion of MCAO. The additional 20 rats were divided into 2 groups of 10 animals each, including the Sham and MCAO (24 h) groups. Tissues from 8 (4 from each group) of the 20 rats were used for immunofluorescence staining to assess the localization of TGR5 in the brain; tissues from the other 12 rats (6 from each group) were used for flow cytometry to evaluate the expression of TGR5 in activated microglia or infiltration by macrophages.

#### Experiment 2

A total of 108 rats was divided into 5 groups: Sham (*n* = 28), MCAO + vehicle (*n* = 34), MCAO + INT777 (0.16 mg/kg, *n* = 6), MCAO + INT777 (0.48 mg/kg, *n* = 34), MCAO + INT777 (1.44 mg/kg, *n* = 6). Based on neurologic tests and brain edema at 24 h after MCAO, the intermediate dosage of INT777 was chosen for ELISA, Western blot analysis at 24 h, neurobehavioral analysis at 72 h, and long-term neurobehavioral assessment.

#### Experiment 3

A total of 18 rats was divided into 3 groups to explore the association between TGR5 and Pellino3 by co-immunoprecipitation (co-IP): Sham (*n* = 6), MCAO + vehicle (*n* = 6), and MCAO + INT777 (0.48 mg/kg, *n* = 6). The immunofluorescence samples used for TGR5 and Pellino3 co-labeling were shared with experiment 1, while the rats used to explore the effect of INT777 on TGR5 and Pellino3 levels (in total protein) were shared with experiment 3.

#### Experiment 4

A total of 168 rats was randomly assigned to 10 groups for a mechanism study: Sham (*n* = 36), MCAO + vehicle (*n* = 36), MCAO + scrambled small interfering RNA (siRNA) (*n* = 12), MCAO + TGR5-siRNA (*n* = 12), MCAO + Pellino3-siRNA (*n* = 12), MCAO + Z-IETD-FMK (*n* = 12), MCAO + INT777 (0.48 mg/kg, *n* = 12), MCAO + INT777 (0.48 mg/kg) + scrambled-siRNA (*n* = 12), MCAO + INT777 (0.48 mg/kg) + TGR5-siRNA (*n* = 12), MCAO + INT777 (0.48 mg/kg) + Pellino3 siRNA (*n* = 12). Neurobehavioral scoring, brain infarction assessments, and Western blot analyses were performed at 24 h (or on samples collected at 24 h) after MCAO.

### Drug administration

To ensure efficient delivery to the CNS, INT777 was administered intranasally 1 h after MCAO, as previously described [13, 23], with some modifications. Specifically, rats received either saline (vehicle), INT777 (0.16 mg/kg), INT777 (0.48 mg/kg), or INT777 (1.44 mg/kg), administered as nose drops (5 μL/drop) over a period of 20 min, alternating drops every 2 min between the left and right nares. The total volume delivered was 50 μL per rat.

Z-IETD-FMK, a caspase-8 inhibitor, was dissolved in sterile DMSO and was delivered at a dose of 1 mg/kg via intravenous (tail vein) injection immediately after MCAO [20].

### Intracerebroventricular siRNA Injection

Three different formats of TGR5 siRNA or Pellino3 siRNA (OriGene Technologies) were diluted with transfection reagent (Entranser™; Engreen Biosystem, Ltd.) and administered, 48 h before MCAO, by intracerebroventricular (ICV) injection as previously described [24, 25]. The TGR5 siRNA, Pellino3 siRNA, scrambled-siRNA mixture (100 pmol in 5 μL) was delivered into the ipsilateral ventricle using a Hamilton microsyringe under the guidance of a stereotaxy instrument. The stereotactic ICV injection site was defined relative to the bregma: posterior 1 mm, right lateral 1.5 mm, depth 3.5 mm. The injection was administered over 5 min, and the needle was left in place for an additional 5 min after injection to prevent possible leakage and then was withdrawn slowly (over 4 min). After the needle was removed, the burr hole was sealed with bone wax. The incision was closed with sutures, and the rat was allowed to recover.

### Neurologic scores

Neurologic examinations were performed by a blinded investigator at 24 h or 72 h after MCAO, as previously described [26]. The assessments consisted of 7 tests covering spontaneous activity, symmetry in limb movement, symmetry of forelimb outstretching, climbing, body proprioception, response to vibrissae touch, and beam walking. The neurologic scoring system ranged from 3 (most severe deficits) to 21 (normal).

### 2,3,5-Triphenyltetrazolium chloride staining

Infarction volume was determined by staining with triphenyltetrazolium chloride (TTC) (Sigma) after MCAO, as previously described [27]. The possible interference of brain edema on infarct volume was corrected by standard methods (whole contralateral hemisphere volume–nonischemic ipsilateral hemisphere volume), and the infarct volume was expressed as a ratio to the whole brain volume [28].

### Immunofluorescent staining

Double- and triple-immunofluorescence staining of the rat brains was performed on fixed frozen ultrathin sections, as previously described [13, 29]. Sequential coronal slices (10-μm thick) were obtained with a cryostat (CM3050S; Leica Microsystems, Wetzlar, Germany) and permeabilized with 0.3% Triton X-100 in phosphate buffer solution (PBS) for 30 min. Sections were blocked with 5% donkey serum for 1 h and incubated overnight at 4 °C with primary antibodies, including: rabbit polyclonal anti-TGR5 (1:100; ab72608; Abcam, Cambridge, MA,USA), mouse monoclonal anti-Pellino3 (1:100; sc-376466; Santa Cruz Biotechnology, Santa Cruz, CA), mouse polyclonal anti-NeuN (1:200; ab104224; Abcam, Cambridge, MA,USA), goat polyclonal anti-glial fibrillary acidic protein (GFAP) (1:200; ab53554; Abcam, Cambridge, MA,USA), and goat polyclonal anti-ionized calcium binding adaptor molecule 1 (Iba-1) (1:200; ab107159; Abcam, Cambridge, MA,USA). Sections then were incubated with the appropriate fluorescence dye-conjugated secondary antibodies (Jackson Immunoresearch, West Grove, PA) in a dark room for 2 h at room temperature. The slices were visualized with a fluorescence microscope (DMi8; Leica Microsystems, Germany) or a confocal Zeiss LSM 710 microscope, and fluorescence intensity was quantified using ImageJ software (Image J 1.4, NIH, USA). Three sections were chosen from each brain, with each section containing three microscopic fields from the ischemic boundary zone.

### Assessment of long-term neurobehavior

From day 21 to day 27 after MCAO, animals were tested using a Morris water maze, as previously described [30]. Additionally, an accelerating rotarod test was used to provide an index of fore- and hindlimb motor coordination and balance [31].

### Evaluation of TNF-α, IL-1β, and IL-18 levels

Twenty-four hours after MCAO, rats were sacrificed, and brain tissue homogenates were obtained from the infarct cerebral hemisphere. The levels of TNF-α,interleukin-1β (IL-1β) and interleukin-18 (IL-18) were measured in brain tissue homogenates using cytokine-specific ELISA kits according to the manufacturers’ instructions; results were quantified using a microplate reader at 450 nm [32].

### Cell isolation and flow cytometry

Twenty-four hours after stroke, the rat brain was isolated and subjected to mechanical and enzymatic dissociation using a tissue dissociation kit (Miltenyi Biotec), as previously described [33, 34]. Suspensions of single cells were mixed with a Percoll suspension and centrifuged at 950 g for 30 min at room temperature. Cells were resuspended in PBS containing 2% BSA and were incubated with the respective antibodies at 4 °C:anti-CD45 (#202207; Biolegend, San Diego, CA,USA), anti-CD11b (#201805; Biolegend, San Diego, CA,USA), and anti-TGR5 (ab72608; Abcam Cambridge, MA,USA). BD CellQuest Pro software (5.1) (CA, USA) was used to determine immune subpopulations. Cells were then gated for CD45-high and CD45-intermediate populations. An Alexa Fluor®488-labeled donkey anti-rabbit IgG secondary antibody was used for the final detection. Data were analyzed using Flow Jo 7.6.1 software (Tree Star, US).

### Western blot analysis

Brain samples were collected 24 h after MCAO. Western blotting was performed as described previously [35]. The proteins of right hemispheres were extracted by cytoplasmic extraction reagents (Pierce Biotechnology, Rockford, IL, USA). Equal amounts of protein (50 μg) were loaded and subjected to electrophoresis on an SDS-PAGE gel. After being transferred to a nitrocellulose membrane, the membranes were cut into different strips which covered an area with target protein according to molecular weight marker location and were blocked with 5% nonfat milk (Bio-Rad Laboratories, Irvine, CA, USA). The membrane was incubated with the primary antibody overnight at 4 °C. Primary antibodies included the following: anti-TGR5 (1:1000; ab72608; Abcam, Cambridge, MA,USA), anti-Pellino3 (1:1000; Santa Cruz Biotechnology, Santa Cruz, CA), anti-caspase-8 (1:1000; ab25901; Abcam, Cambridge, MA,USA), anti-NLRP3 (1:500; NBP2-12446; NOVUS, CO, USA), anti-caspase-1 (1:1000; NBP1-45433; NOVUS, CO, USA), and IL-1β (1:1000; ab2105; Abcam, Cambridge, MA, USA). β-actin (1:1000, sc-58673, Santa Cruz, TX, USA) was used as an internal loading control. The secondary antibodies were all from Santa Cruz Biotechnology. Blot bands were quantified by densitometry using ImageJ software (ImageJ 1.4, NIH, USA).

### Co-immunoprecipitation

Co-immunoprecipitation (Co-IP) was performed as previously described [36]. An aliquot of 500 μg total protein was first pre-treated with polyclonal anti-TGR5 antibody (1:50) or anti-Pellino3 (1:50) with agitation on a rotator. Protein A/G agarose (20 μL; Sigma) was added to each sample, and the mixture was incubated overnight at 4 °C. The mixture was then precipitated by high-speed freezing centrifugation at 12,000 rpm for 10 s. To remove non-specifically bound proteins, the pellet was washed three times with NP-40 buffer. Agarose-bound immunocomplexes then were released by resuspension in a loading buffer containing denaturing solution. The resulting suspensions were electrophoresed and used for Western blot analysis as described above, probing with antibodies against TGR5 and Pellino3 proteins. For comparison, samples of total protein also were run on the same gels.

### Statistical analysis

Statistical analyses of the data were performed using SigmaPlot 11.0 and Prism 6 (GraphPad, San Diego, CA). Data are presented as mean standard deviation (SD), where appropriate. Data from different groups were compared using two-tailed One-way ANOVA followed by post hoc Tukey’s tests. Non-parametric data (neurological scores, beam walking) were analyzed using a two-tailed Kruskal–Wallis test followed by post hoc Dunn’s tests. No further adjustment for the overall number of tests was made when performing multiple comparisons. For all statistical analyses, a value of *P* < 0.05 was considered statistically significant.

## Results

### Mortality and exclusions

A total of 397 rats was used, of which 311 underwent MCAO induction. The mortality of MCAO rats was 13.8% (43 of 311) in our hands; no rats died in the other groups (Supplemental Table 1). Ten animals were excluded; the excluded rats included animals that did not show signs of neurobehavioral deficits when waking up from MCAO (body twisting when lifted by the tail and walking in circles) or animals in which subarachnoid hemorrhage was observed at necropsy.

### The level of endogenous TGR5 receptor increases after MCAO

**Fig. 1:**
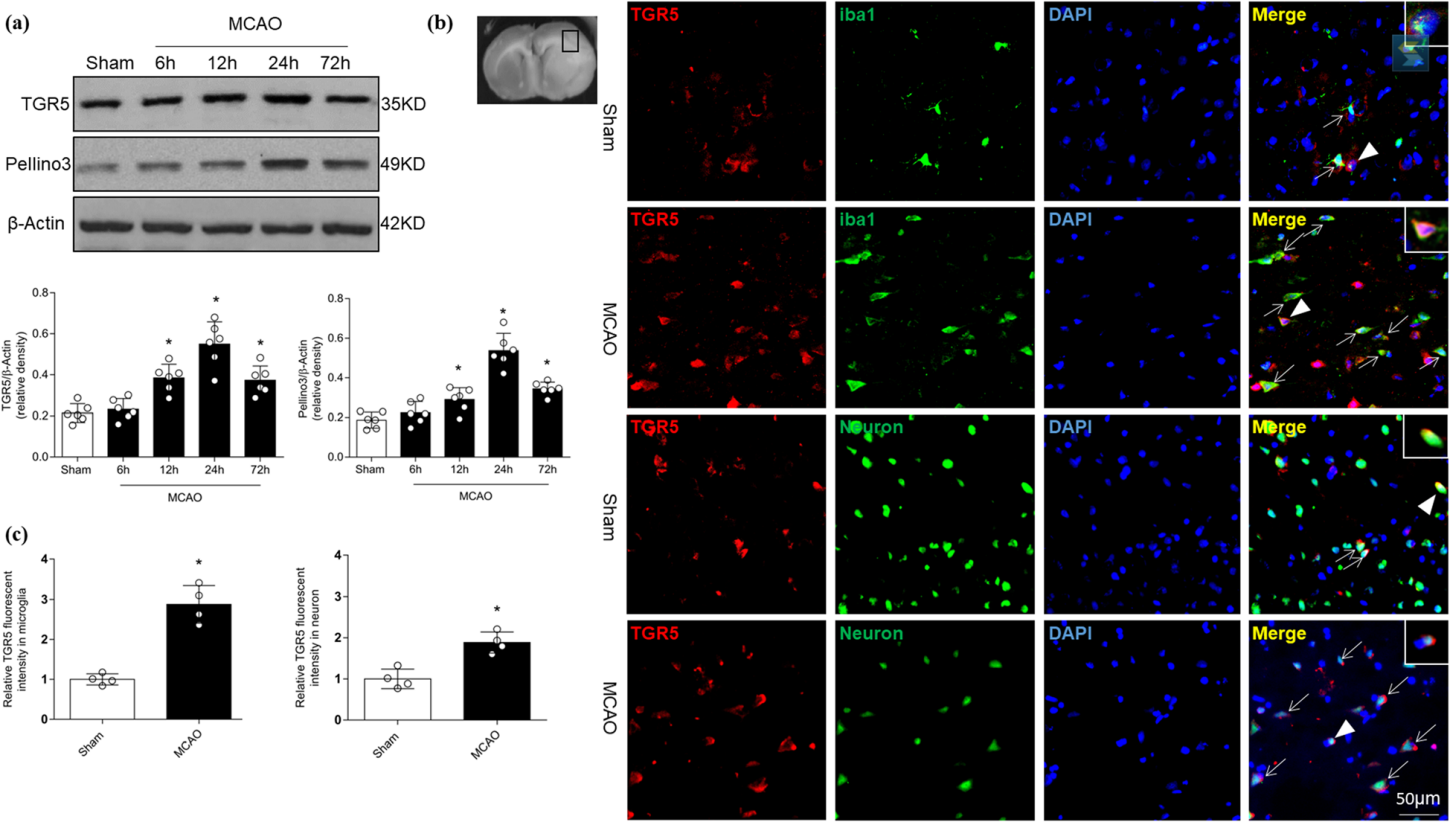
TGR5 and Pellino3 levels after middle cerebral artery occlusion (MCAO). **a** Representative Western blot images and quantitative analyses of time courses of TGR5 and Pellino3 proteins from right hemisphere after MCAO. *n* = 6 per group, *P < 0.05 vs. Sham group. **b** Double immunofluorescence staining for TGR5 (red) in microglia (Iba-1, green), neuron (green) in the penumbra following MCAO. *n* = 4 per group. **c** The relative fluorescent intensity of TGR5 in microglia and neurons, *n* = 4 for each group, *P < 0.05 vs. Sham. Bars represent mean ± SD. Scale bar, 50 μm. Iba-1, ionized calcium binding adaptor molecule 1

As shown in Fig. 1a, TGR5 protein levels were significantly increased at 12 h after MCAO, peaking at 24 h before declining by 72 h after MCAO (*P* < 0.05 vs. Sham). The level of Pellino3 protein was elevated at 12 h after MCAO, again peaking at 24 h before decreasing significantly at 72 h after MCAO (Fig. 1a; *P* < 0.05 vs. Sham).

**Fig. 2:**
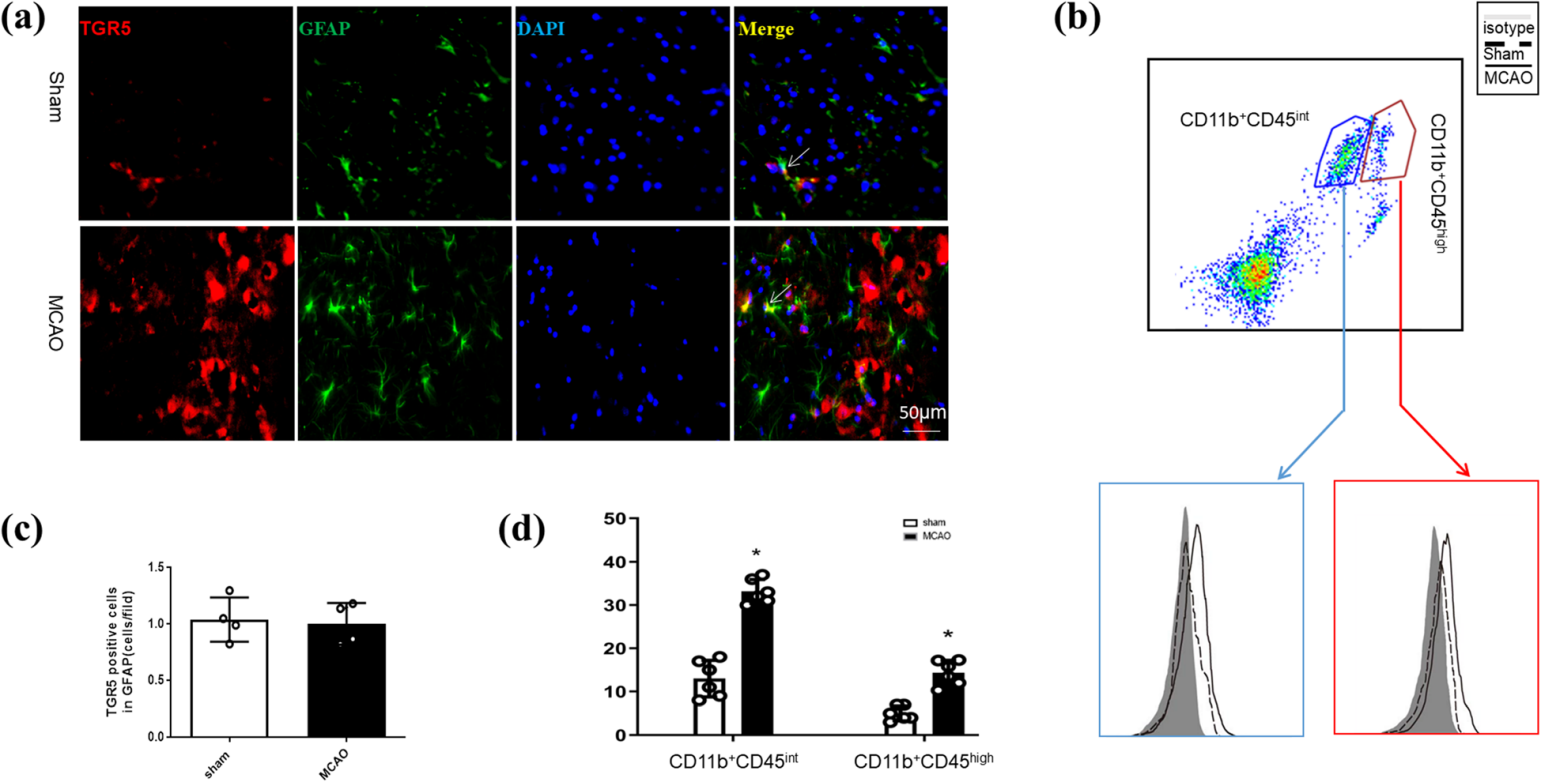
Expression of TGR5 after middle cerebral artery occlusion (MCAO). **a**, **b** Double immunofluorescence staining and relative fluorescent intensity of TGR5 (red) in GFAP (green) in the penumbra following MCAO. *n* = 4 per group, *P < 0.05 vs. Sham. Scale bar, 50 μm. **c**, **d** Flow cytometry showing that TGR5 accumulates to higher levels in CD11b^+^CD45^intermediate^ microglia and CD11b^+^CD45^high^ macrophages at 24 h after MCAO. *n* = 6 for each group, *P < 0.05 vs. Sham. GFAP, glial fibrillary acidic protein

Double immunofluorescence staining showed that TGR5 accumulated at higher levels in microglia and neurons in the penumbral area 24 h after MCAO (Fig 1b, c). No significant differences were seen in astrocytic TGR5 expression between he Sham and MCAO groups (Fig. 2a, b). After stroke was induced, TGR5 levels were increased in CD11b^+^CD45^intermediate^ microglia and CD11b^+^CD45^high^ macrophages compared with TGR5 levels in the respective cells before stroke (Fig. 2c, d). Simultaneously, Pellino3 expression was also observed in microglia or neurons in the penumbral area following MCAO (Supplemental Figure 2).

### TGR5 receptor agonist treatment reduces brain infarction and improves neurological function at 24 and 72 h after MCAO

**Fig. 3:**
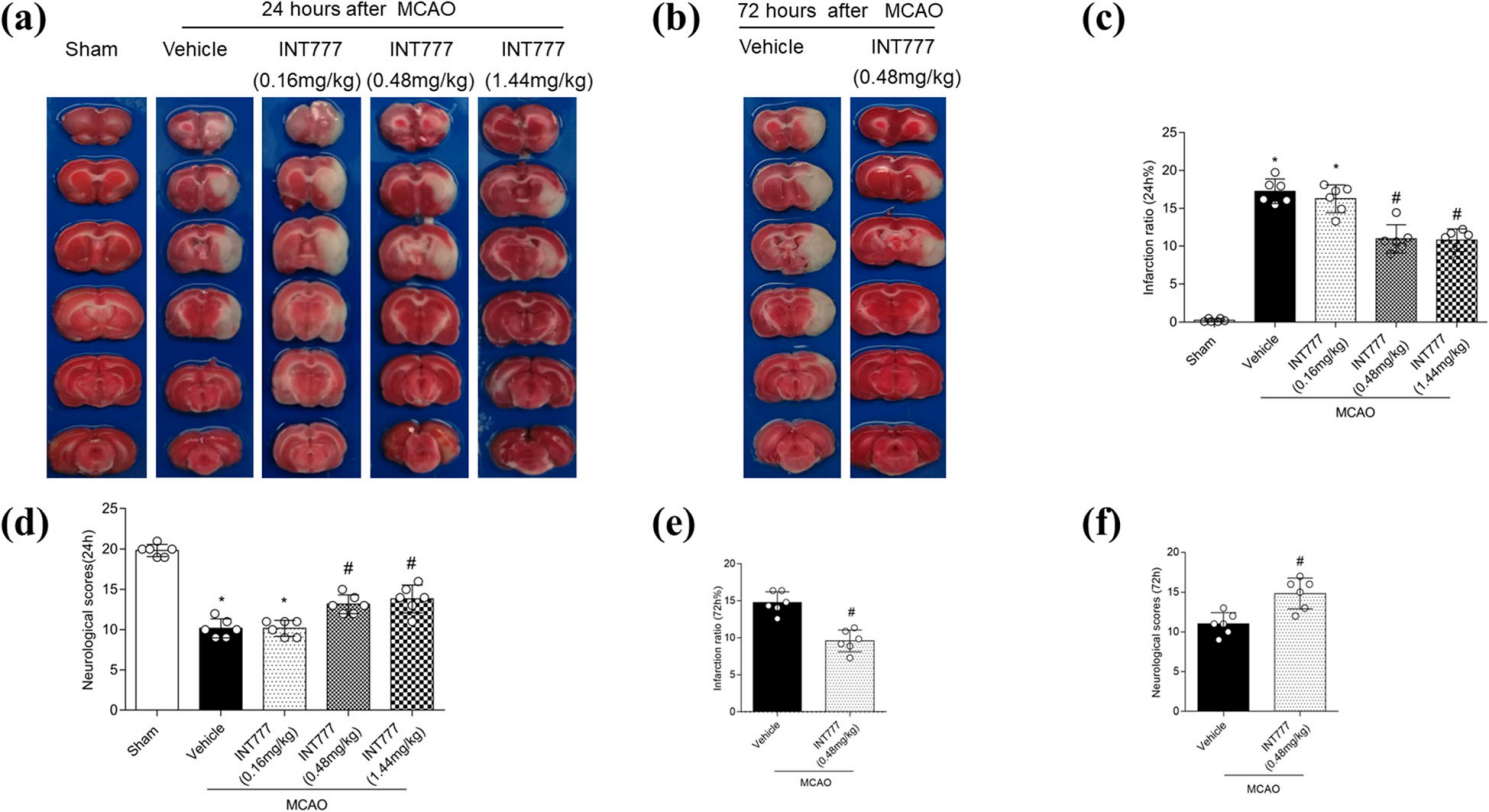
The protective role of TGR5 in following middle cerebral artery occlusion (MCAO). **a**, **b** INT777 ameliorates brain injuries at both 24 and 72 h after MCAO. Representative TTC staining images of coronal sections. **c**–**f** Quantified infarct ratio, neurological scores. *n* = 6 for each group. *P < 0.05 vs. Sham, ^#^*P* < 0.05 vs. MCAO + vehicle. Bars represent mean ± SD

Compared with animals of the MCAO + vehicle group, rats treated with intermediate- (0.48 mg/kg) or high- (1.44 mg/kg) dose INT777 exhibited significantly reduced infarct volume and improved neurological scores (respectively) at 24 h after MCAO (Fig. 3a, c, d; *P* < 0.05). The intermediate dosage of INT777 also decreased cerebral infarction and improved neurological function at 72 h after injury (Fig. 3b, e, f); *P* < 0.05 vs. MCAO + vehicle). Therefore, we chose to use intermediate dose levels for the long-term and mechanistic studies. Notably, MCAO rats treated with the intermediate INT777 dose did not differ from MCAO + vehicle animals regarding respiratory parameters, blood pressure, or body temperature (Supplemental Table 2).

### TGR5 activation improves long-term neurobehavioral outcomes after MCAO

**Fig. 4:**
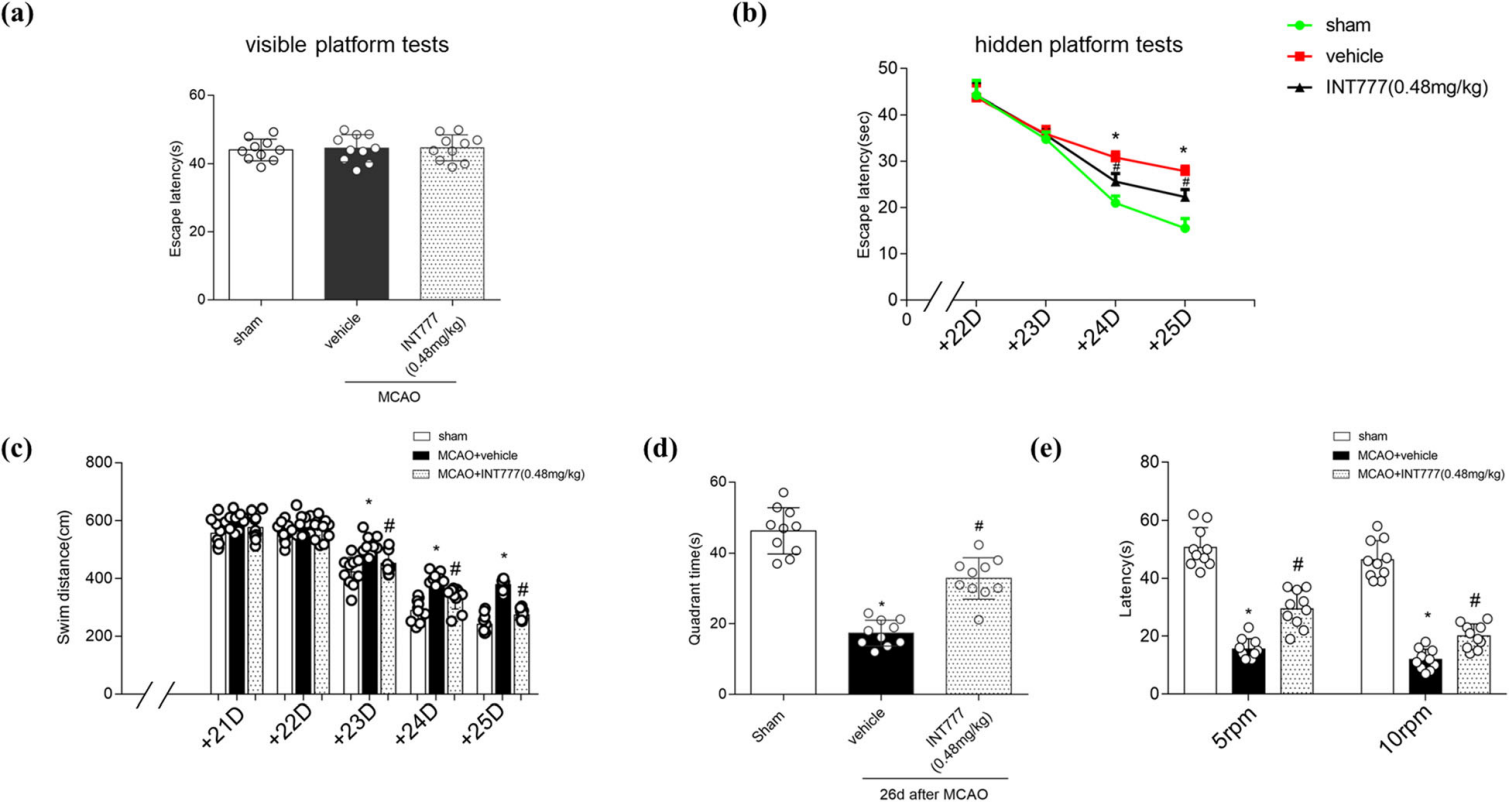
INT777 improves long-term neurobehavior after middle cerebral artery occlusion (MCAO). **a**, **b** Escape latency during the visible platform test and hidden platform tests. **c** Swim distance of water maze test. **d** Probe quadrant duration of water maze test. **e** Rotarod test at 5 RPM and 10 RPM. *n* = 6 for each group. *P < 0.05 vs. Sham, ^#^*P* < 0.05 vs. MCAO + vehicle. Bars represent mean ± SD

We performed Morris water maze and rotarod tests on days 21–27 after MCAO. The three groups exhibited a similar latency to escape to the visible platform and had similar swimming distances during the first day of visible platform tests (Fig. 4a, c; *P* > 0.05). For the hidden platform trials and probe trial, the results showed that the animals in the vehicle group required more time to reach the platform (Fig. 4b; *P* < 0.05), traveled a significantly longer distance (Fig. 4c; *P* < 0.05), and spent less time in the target probe quadrant (Fig. 4d; *P* < 0.05) compared to the Sham group. Dosing with INT777 decreased the latency to finding the platform, reduced the travel distance, and led to animals spending significantly more time in the target quadrant (Fig. 4b–d; *P* < 0.05 vs. MCAO + vehicle).

In the rotarod test, INT777 treatment significantly improved motor coordination on the 5-RPM and 10-RPM tests (Fig. 4e; *P* < 0.05 vs. MCAO + vehicle).

### INT777 suppresses neuroinflammation induced by MCAO

**Fig. 5:**
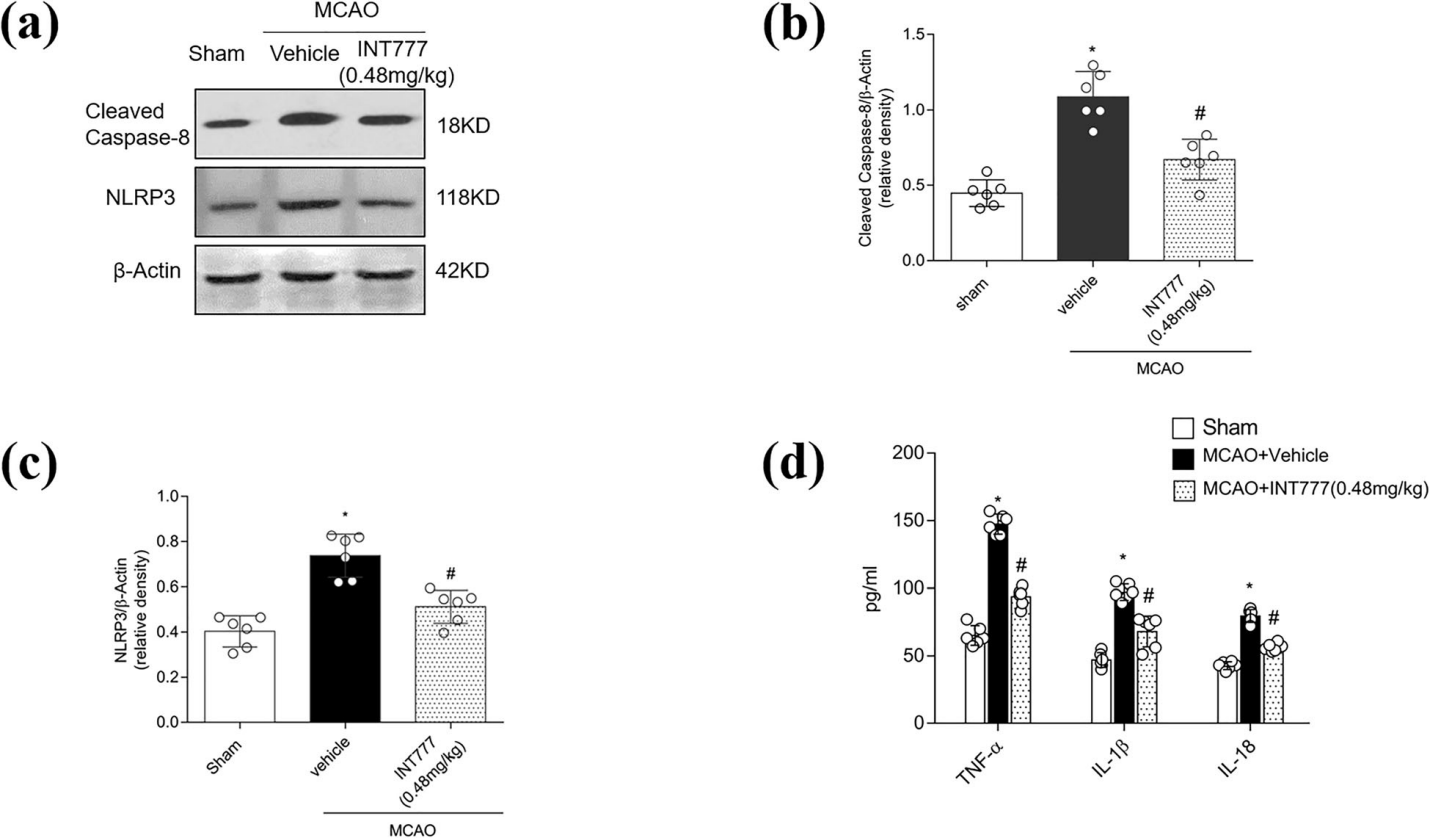
INT777 suppresses neuroinflammation induced by middle cerebral artery occlusion (MCAO). **a**–**c** Representative western blot images and quantitative analysis of cleaved caspase-8 and NLRP3 bands. **d** Effects of INT777 on levels of TNF-α, IL-1β, and IL-18 in brain tissue. *n* = 6 per group. *P < 0.05 vs. Sham, ^#^*P* < 0.05 vs. MCAO + vehicle

At 24 h after MCAO, cleaved caspase-8 and NLRP3 levels were significantly increased (*P* < 0.05 vs. Sham); INT777 treatment led to a depletion of these proteins compared with the levels in untreated MCAO rats (Fig. 5a–c; *P* < 0.05 vs. MCAO + vehicle). The levels of TNF-α, IL-1β, and IL-18 were increased in the MCAO group compared with the Sham group (*P* < 0.05). In the INT777 treatment group, the levels of these cytokines in brain tissue were reduced significantly (*P* < 0.05 vs. MCAO + vehicle, Fig. 5d).

### MCAO induces TGR5 with Pellino3 interactions

**Fig. 6:**
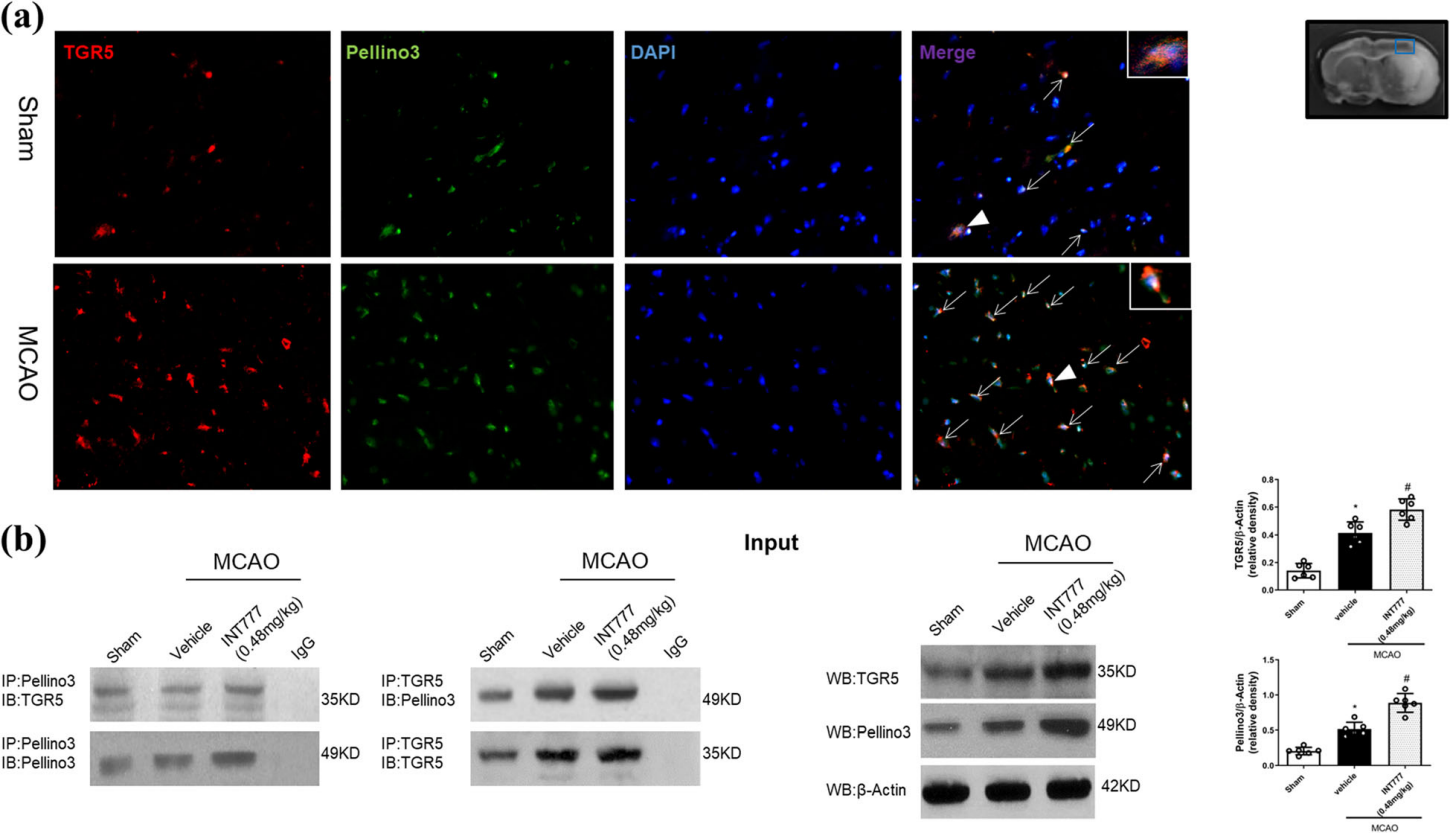
TGR5 interacts with Pellino3 after middle cerebral artery occlusion (MCAO). **a** Double immunofluorescence staining showed that co-localization of TGR5 (red) and Pellino3 (green) is increased in the penumbra 24 h after MCAO. *n* = 4 per group. **b** Representative co-IP bands showed that interactions of TGR5 with Pellino3 occurred at 24 h after MCAO. Protein bands of TGR5 and Pellino3 in total protein solution were detected by Western blot and quantitative analysis are reported in right panel. *n* = 6 for each group. *P < 0.05 vs. Sham, ^#^*P* < 0.05 vs. MCAO + vehicle. Bars represent mean ± SD

In the Sham group, double immunofluorescence staining showed that TGR5 and Pellino3 co-localized in the brain. After MCAO, co-labeling of TGR5 with Pellino3 increased in the penumbral area (Fig. 6a). Triple-fluorescence staining showed that TGR5 and Pellino3 co-localized in microglia (Supplemental Fig. 3a).

Western blot analysis showed that the levels of both TGR5 and Pellino3 increased after MCAO (Fig. 6b; P < 0.05 vs. Sham). Co-IP showed that the TGR5-Pellino3 interaction was found in the ischemic hemisphere (Fig. 6b).

### Dosing with Pellino3 siRNA increases the accumulation of cleaved caspase-8 and NLRP3 after MCAO

Firstly, we explored the relationship between caspase-8 and NLRP3 expression. Administration of the caspase-8 inhibitor Z-IETD-FMK resulted in a depletion of NLRP3. Pellino3 siRNA significantly decreased Pellino3 protein levels at 24 h after MCAO. Western blotting showed that Pellino3 knockdown significantly increased expression of both cleaved caspase-8 and NLRP3 (Supplemental Fig. 3b, c; *P* < 0.05 vs. MCAO + vehicle). Pellino3 siRNA significantly increased infarct volumes and aggravated the neurologic scores, while treatment with Z-IETD-FMK counteracted neurologic damage (Supplemental Fig. 3d; *P* < 0.05 vs. MCAO + vehicle).

### TGR5 or Pellino3 knockdown prevents the anti-inflammatory activity of INT777 after MCAO

**Fig. 7:**
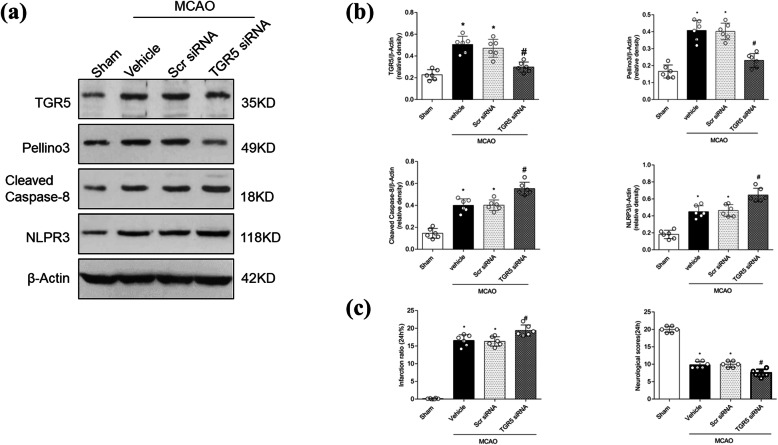
The effect of TGR5 knockdown on the levels of Pellino3, cleaved caspase-8, and NLRP3, and on brain damage after middle cerebral artery occlusion (MCAO). The band of Western blot analysis (**a**) and the relative density (**b**) of TGR5, Pellino3, cleaved caspase-8, and NLRP3. **c** Dosing with TGR5 siRNA increases infarct volume and worsens neurobehavioral deficits. *n* = 6 per group. *P < 0.05 vs. Sham, ^#^*P* < 0.05 vs. MCAO + Scr-siRNA. Bars represent mean ± SD. *Scr*-*siRNA* scrambled-siRNA

Compared with the vehicle group, the TGR5 knockdown group showed decreases in TGR5 and Pellino3 levels along with increases in cleaved caspase-8 and NLRP3 levels (Fig. 6a, b; *P* < 0.05). TGR5 siRNA significantly increased brain infarct volumes and neurologic impairments (Fig. 7c; *P* < 0.05 vs. scrambled-siRNA group).

**Fig. 8:**
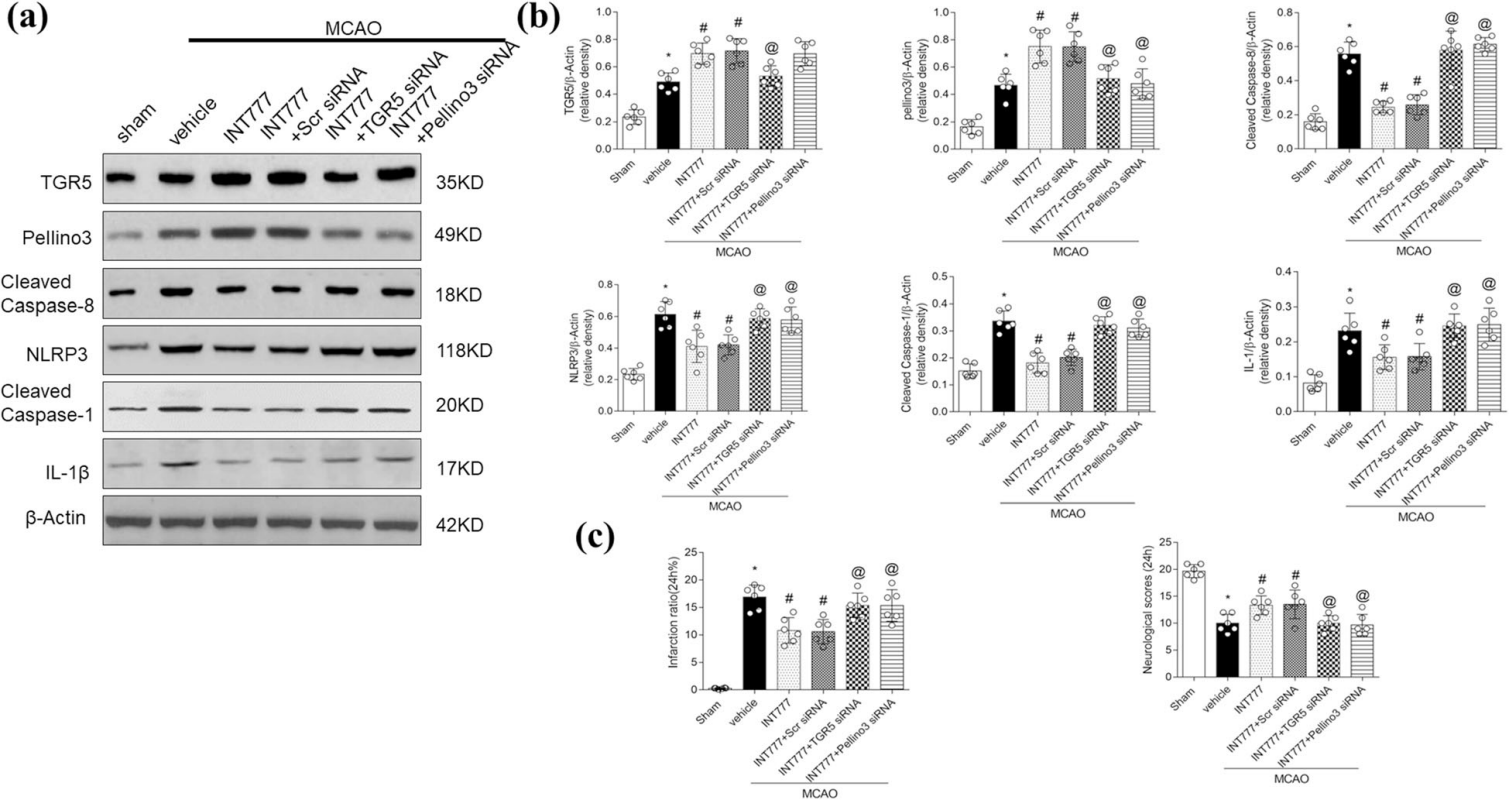
TGR5 or Pellino3 knockdown abolishes the anti-inflammatory effects of INT777 after middle cerebral artery occlusion (MCAO). **a**, **b** Representative Western blot bands and quantitative analyses of the protein levels of TGR5, Pellino3, cleaved caspase-8, NLRP3, cleaved caspase-1, and IL-1β. **c** Quantified infarct ratio and neurological scores, *n* = 6 per group. *P < 0.05 vs. Sham, ^#^*P* < 0.05 vs. MCAO + vehicle, @*P* < 0.05 vs. MCAO + INT777 + Scr-siRNA group. *Scr siRNA* scrambled-siRNA

TGR5 siRNA reversed the effect of INT777 on the accumulation of Pellino3, cleaved caspase-8, and NLRP3 (Fig. 8a, b; *P* < 0.05 vs. MCAO + INT777 + scrambled-siRNA). Western blotting showed that dosing with Pellino3 siRNA also significantly reversed the effect of INT777 on the levels of cleaved caspase-8 and NLRP3, as well as effects on cleaved caspase-1 and IL-1β levels compared with the effect of scrambled-siRNA at 24 h after MCAO (Fig. 8a, b; *P* < 0.05).

Administration of TGR5 siRNA or Pellino3 siRNA significantly attenuated the neurological improvements associated with treatment with INT777 at 24 h after MCAO (Fig. 8c; *P* < 0.05 vs. MCAO + INT777 + scrambled siRNA).

## Discussion

In this study, we investigated the role of TGR5 in counteracting inflammation following MCAO. This study demonstrated that TGR5 and Pellino3 accumulate to higher levels in the injured hemisphere after MCAO. Treatment with INT777 improved both short- and long-term neurofunction after MCAO, effects that were accompanied by increases in Pellino3 levels, decreases in pro-inflammatory cytokines levels, and depletion of cleaved caspase-8 and NLRP3 levels. In contrast, knockdown of endogenous TGR5 or Pellino3 by siRNA increased cleaved caspase-8 and NLRP3 levels, and exacerbated brain injury. Furthermore, knockdown of TGR5 or Pellino3 abolished the anti-neuroinflammatory effects of INT777. Taken together, these results suggest that TGR5 could be involved in inhibiting neuroinflammation in the brain, an effect that appears to be mediated via Pellino3 the inhibition of caspase-8/NLRP3 accumulation after MCAO in rats.

Previous studies have suggested a protective role for TGR5 agonists in inflammation. Research shows that TGR5 might suppress gastric, liver, and renal inflammation [37–39]. Furthermore, Yang et al. found that TGR5 inhibited expression of inflammatory cytokines in liver ischemia [21]. Upregulation of TGR5 was observed in the cortex of mice with hepatic encephalopathy; central infusion of a TGR5 agonist delayed neurological decline [12]. It has been reported that TGR5 is constitutively expressed in microglia, both in vivo and in vitro [40]. Binding of the bile acid tauroursodeoxycholic acid (TUDCA) to TGR5 causes an increase in intracellular cyclic adenosine monophosphate (cAMP) levels in microglia, leading to the production of anti-inflammatory markers while decreasing the levels of pro-inflammatory cytokines [40]. INT777 (6α-ethyl-23(S)-methyl-cholic acid; 6-EMCA) is a specific TGR5 agonist without farnesoid X receptor activity; research has shown that intracerebroventricular injection of INT777 significantly improves amyloid-beta (Aβ) 1–42-induced cognitive impairment [41]. There is growing evidence that intranasal administration is a viable route for delivery to the brain to treat neuroinflammation [23]. Indeed, the results of mass spectrometric analysis have shown that this route of administration is sufficient to provide an effective brain concentration of INT777 [42]. Several studies have found that intranasal administration of INT777 reduces neuroinflammation and improves cognitive impairment in experimental rat models of sepsis and of neurological deficit after subarachnoid hemorrhage [42, 43]. In the present study, we observed that intranasal administration of INT777 significantly decreases the accumulation of NLRP3 and improves neurobehavioral functions after MCAO. In addition, TGR5 siRNA aggravated brain impairments in MCAO animals, counteracting the neuroprotective effects of INT777.

Although the exact mechanisms of TGR5-mediated neuroprotection are not well understood, Pellino3 might play a critical role in the TGR5-mediated signaling pathway. Pellino3 belongs to the mammalian Pellino family of E3 ubiquitin ligases that play important roles in innate immunity [44, 45]. Pellino3-deficient mice show heightened diet-induced inflammation and increased IL-1β levels, resulting in exacerbation of insulin resistance [15]. Smith et al. found that Pellino3 decreased TLR2-mediated NF-κB activity and the expression of genes encoding proinflammatory proteins in epithelial cells following exposure to *Helicobacter pylori* LPS [46]. In the present study, we observed that endogenous Pellino3 levels were increased 24 h after MCAO, and that INT777 further enhanced the accumulation of Pellino3 protein. Double immunofluorescence staining demonstrated that co-localization of TGR5 with Pellino3 increased after MCAO. Co-IP also showed an interaction between TGR5 and Pellino3 after MCAO. Furthermore, we observed that silencing of TGR5 counteracted the increase in Pellino3 levels induced by INT777. Taken together, these findings imply that TGR5 acts upstream within a signaling axis to increase the accumulation of Pellino3 protein, thereby alleviating neuroinflammation. Yang et al. found that loss of Pellino3 in mice led to high caspase-8 levels, along with hepatoxicity and lethality, in response to in vivo administration of TNF [16]. In the present work, we found that the level of cleaved caspase-8 was increased after MCAO, consistent with previous observations [47, 48]. Treatment with Pellino3 siRNA resulted in a significant increase in cleaved caspase-8 levels and abolished the effects of INT777 on cleaved caspase-8 accumulation.

Several lines of evidence have confirmed that caspase-8 functions as a regulatory molecule for pro-inflammatory activation of microglia [47, 49]. However, the role of caspase-8 as a regulator of the NLRP3 inflammasome remains controversial. Several studies have suggested that caspase-8 acts upstream of NLRP3 activation. For example, in the absence of an inhibitor of apoptosis, caspase-8 is essential for TLR-mediated, NLRP3-induced caspase-1 processing [50]. Chi et al. found that caspase-8 promoted NLRP3 inflammasome activation and IL-1β production in acute glaucoma [18]. In contrast, studies of dendritic cells harboring a conditional knockout of the caspase-8-encoding locus (*caspase-8*-cKO DCs) implicated caspase-8 as an inhibitor of RIPK3-mediated NLRP3 activation [51]. In the present work, we found that inhibition of caspase-8 activation resulted in significant depletion of NLRP3, an observation that agrees with other work indicating a role for caspase-8 in intraocular pressure-induced retinal ischemia [18]. Furthermore, our data demonstrated that dosing with TGR5 siRNA or Pellino3 siRNA significantly reversed the effect of INT777 on the levels of cleaved caspase-8 and NLRP3 and abolished the neuroprotective activity of INT777. Our findings support the Pellino3/caspase-8/NLRP3 signaling pathway as part of the underlying neuroprotective mechanism of TGR5 activation following MCAO.

This study has several limitations. Firstly, TGR5 has been shown to interact with other membrane receptors [52]; therefore, there could be additional mechanisms or interactions that play a role in TGR5 neuroprotection. Secondly, there could be cross-talk between the downstream signaling molecules that might also play a role in the observed protection; assessments at earlier time points, which could clarify downstream and upstream interactions among the relevant pathways, should be employed in future studies. Thirdly, the protective effects of INT777 could be direct (i.e., via TGR5 signaling) but also could include indirect effects mediated by the reduction of the infarction. It is very likely that reducing infarction will reduce pro-inflammatory effects. Fourthly, although INT777 was protective in the present research, and we cannot exclude the possibility that other TGR5 agonists might extend the time for treatment. Finally, Wu et al. have demonstrated that INT777 counteracts Aβ_1-42_-induced cognitive impairment via suppression of apoptosis [41], and other work has shown that activation of TGR5 markedly attenuates hypoxia/reoxygenation-induced hepatocellular apoptosis [21]. We cannot exclude the possibility that TGR5 activation decreases infarct volume by reducing neuronal death, a hypothesis that will need to be explored in further studies.

## Conclusions

In summary, the present study showed that TGR5 accumulates following MCAO and that treatment with an exogenous TGR5 agonist inhibits neuroinflammation and improves the neurological outcome. The effect of this agonist might be mediated via Pellino3 inhibition of caspase-8/NLRP3 accumulation after MCAO in rats. These observations suggest that TGR5 could be an attractive candidate target for the development of clinical treatments to reduce neuroinflammation following stroke.

## Supplementary Information

**Additional file 1: Figure S1**. Experimental design and animal group classification. IF, immunofluorescence; icv, intracerebral ventricular; MCAO, middle cerebral artery occlusion; Scr siRNA, Scramble small interfering RNA; WB, western blot; Co-IP, Co-immunoprecipitation

**Additional file 2: Figure S2**. Expression of Pellino3 after middle cerebral artery occlusion (MCAO). Double immunofluorescence staining for Pellino3 (red) in microglia (Iba-1, red), neuron (red) in the penumbra following MCAO. n=4 per group

**Additional file 3: Figure S3**. a Triple-fluorescence staining showed that TGR5 and pellino3 colocalized in the microglia after middle cerebral artery occlusion (MCAO), n=4 per group. Scale bar, 10 μm. b,c Effect of Pellino3 siRNA and caspase-8 inhibitor on NLRP3 expression after MCAO, Western blot analysis and relative density. n=6 per group. *P<0.05 vs sham, ^#^*P*<0.05 vs MCAO+ vehicle. Scr siRNA, Scramble small interfering RNA. d Effect of Pellino3 siRNA and caspase-8 inhibitor on infarct volume and neurobehavioral deficits. n=6 for each group. *P<0.05 vs sham, ^#^*P*<0.05 vs MCAO + vehicle. Bars represent mean±SD

**Additional file 4: Table S1**. Animal number (Survival/total) in each group. **Table S2.** Physiological parameters

## Data Availability

The datasets used and/or analyzed in the current study are available from the corresponding authors on reasonable request.

## Abbreviations

Aβ: Amyloid-beta
BSA: Bovine serum albumin
cAMP: Cyclic adenosine monophosphate
CCA: Common carotid artery
CNS: Central nervous system
Co-IP: Co-immunoprecipitation
GFAP: glial fibrillary acidic protein
ECA: External carotid artery
Iba-1: Ionized calciumbinding adaptor molecule 1
ICA: Internal carotid artery
ICV: Intracerebroventricular
IL-1β: Interleukin-1β
IL-18: Interleukin-18
LPS: Lipopolysaccharide
MCAO: Middle cerebral artery occlusion
NLRP3: Nucleotide-binding oligomerization domain-like receptor pyrin domain-containing protein 3
PBS: Phosphate buffer solution
siRNA: Small interfering RNA
TGR5: Trans-membrane G protein-coupled receptor-5
TNFα: Tumor necrosis factor-α
TTC: Triphenyltetrazolium chloride
TUDCA: Tauroursodeoxycholic acid
