# Supplementary figures and images for: TGR5 activation attenuates neuroinflammation via Pellino3 inhibition of caspase-8/NLRP3 after middle cerebral artery occlusion in rats
Source: J Neuroinflammation. 2021 Feb 2;18:40. doi: 10.1186/s12974-021-02087-1 (PMC7856773; doi:10.1186/s12974-021-02087-1)

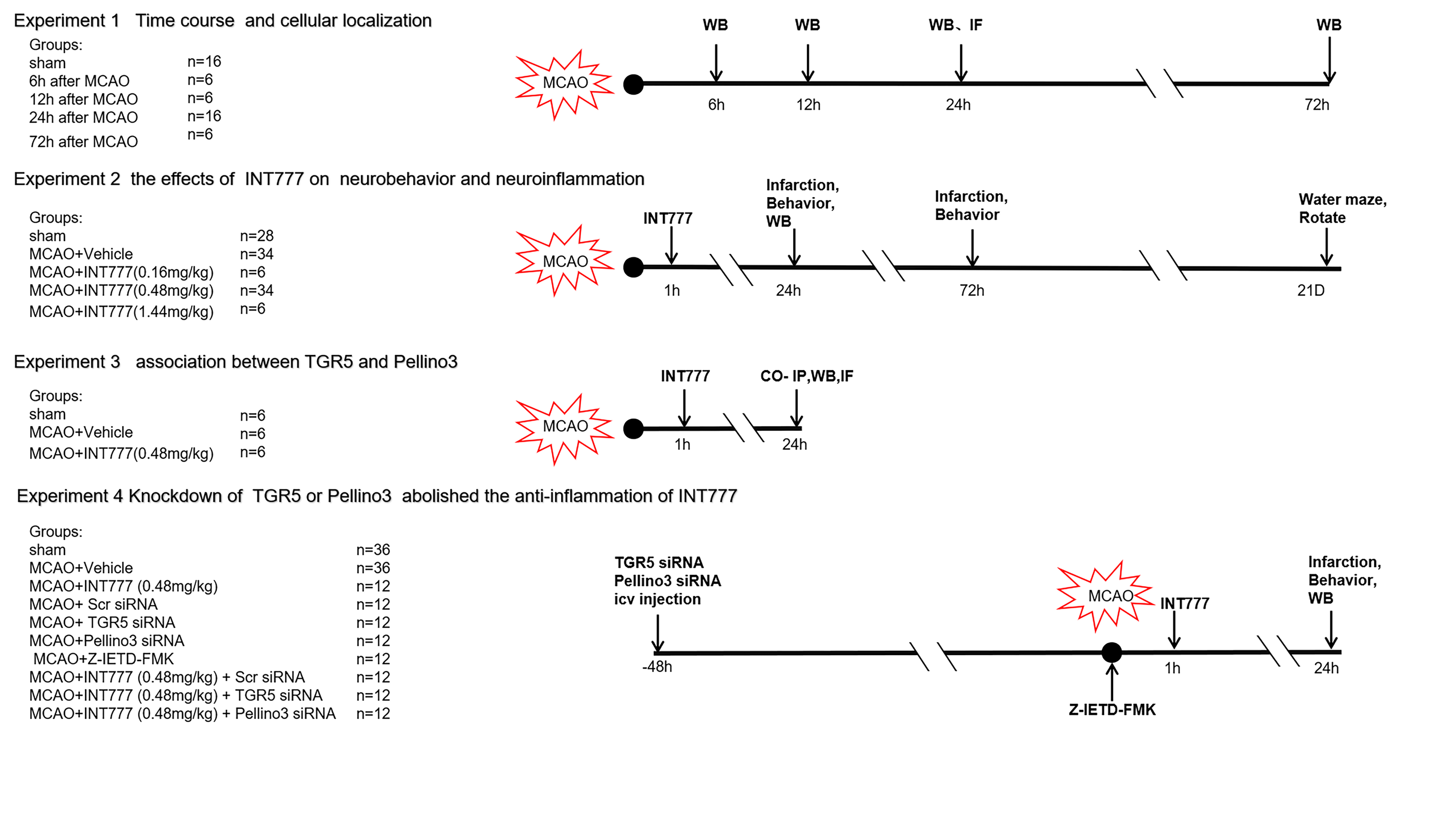

Supplement: Supplementary file 1 — Additional file 1: Figure S1. Experimental design and animal group classification. IF, immunofluorescence; icv, intracerebral ventricular; MCAO, middle cerebral artery occlusion; Scr siRNA, Scramble small interfering RNA; WB, western blot; Co-IP, Co-immunoprecipitation [file 12974_2021_2087_MOESM1_ESM.tif]

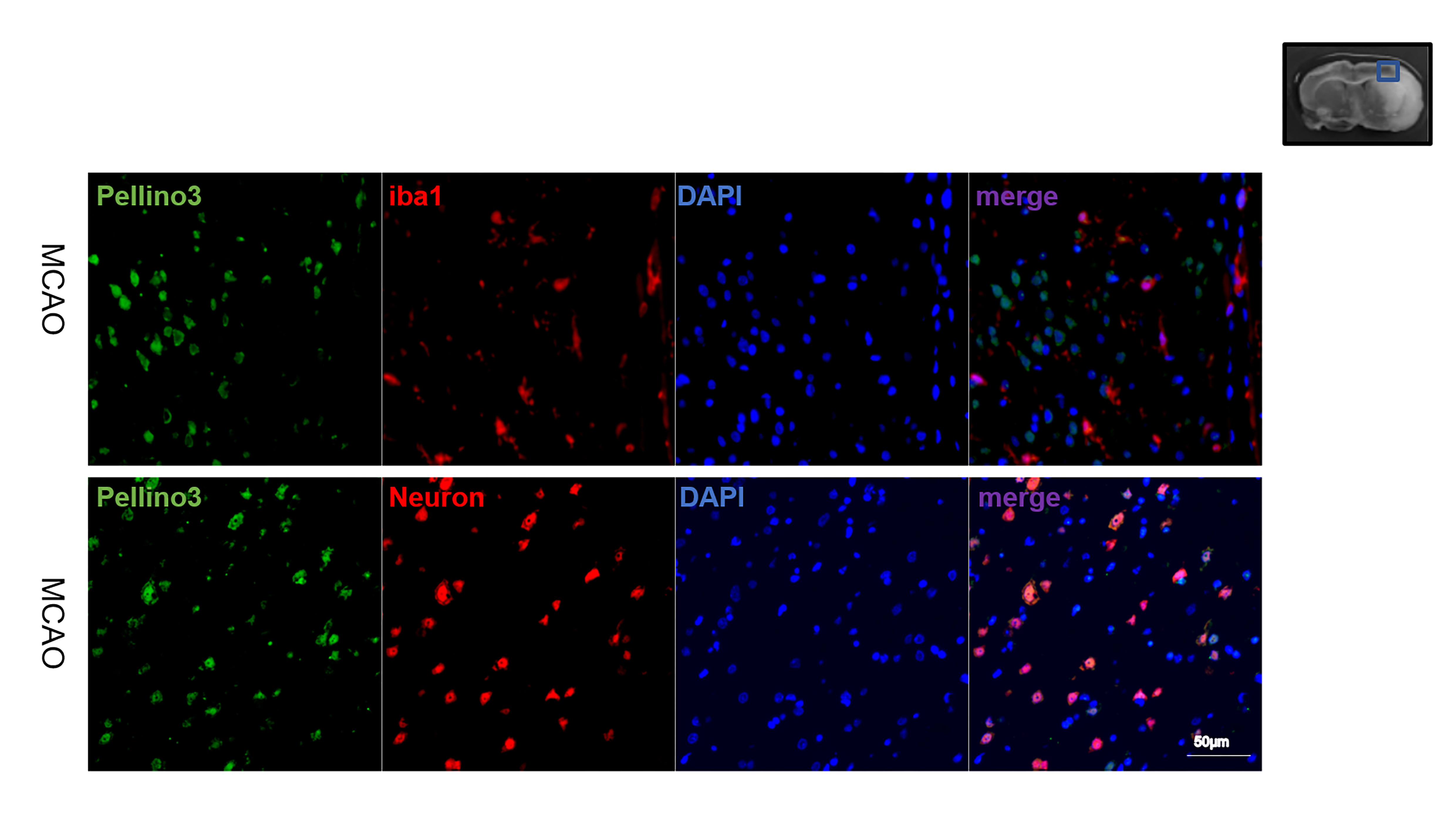

Supplement: Supplementary file 2 — Additional file 2: Figure S2. Expression of Pellino3 after middle cerebral artery occlusion (MCAO). Double immunofluorescence staining for Pellino3 (red) in microglia (Iba-1, red), neuron (red) in the penumbra following MCAO. n=4 per group [file 12974_2021_2087_MOESM2_ESM.tif]

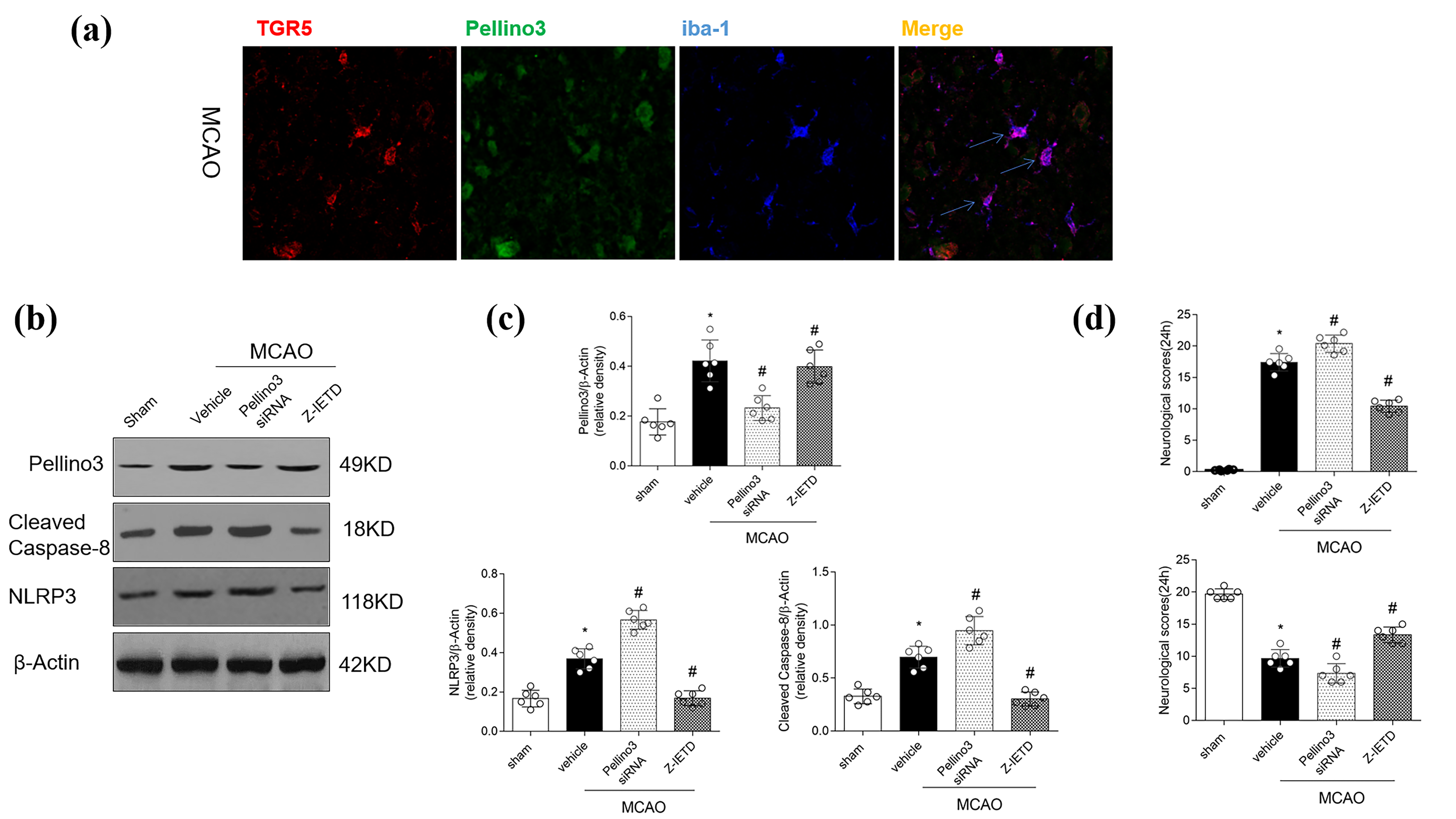

Supplement: Supplementary file 3 — Additional file 3: Figure S3. a Triple-fluorescence staining showed that TGR5 and pellino3 colocalized in the microglia after middle cerebral artery occlusion (MCAO), n=4 per group. Scale bar, 10 μm. b,c Effect of Pellino3 siRNA and caspase-8 inhibitor on NLRP3 expression after MCAO, Western blot analysis and relative density. n=6 per group. *P<0.05 vs sham, #P<0.05 vs MCAO+ vehicle. Scr siRNA, Scramble small interfering RNA. d Effect of Pellino3 siRNA and caspase-8 inhibitor on infarct volume and neurobehavioral deficits. n=6 for each group. *P<0.05 vs sham, #P<0.05 vs MCAO + vehicle. Bars represent mean±SD [file 12974_2021_2087_MOESM3_ESM.tif]
