# Supplementary material for: TGR5 activation attenuates neuroinflammation via Pellino3 inhibition of caspase-8/NLRP3 after middle cerebral artery occlusion in rats
Source: J Neuroinflammation. 2021 Feb 2;18:40. doi: 10.1186/s12974-021-02087-1 (PMC7856773; doi:10.1186/s12974-021-02087-1)
Supplement: Supplementary file 4 — Additional file 4: Table S1. Animal number (Survival/total) in each group. Table S2. Physiological parameters [file 12974_2021_2087_MOESM4_ESM.docx]

**Supplemental material
 Full title:** TGR5 activation attenuates neuroinflammation via Pellino3 inhibition of

Caspase-8/NLRP3 after middle cerebral artery occlusion in rats

**Authors’ names:** Hui Liang, Nathanael Matei， Devin W. McBride, Yang Xu, Zhenhua Zhou, Jiping Tang, Benyan Luo, John H. Zhang

**Supplemental Methods**1. Sample size and power calculations
The sample size to be estimated for all groups with formula: sample size n=Z * Z [P (1-P)/(D*D)]. P=Expected Frequency Value=80%. Z=1.960 with Confidence Level of 95%. D = (Expected Frequency - Worst Acceptable) = 0.25. Sample size estimates were then made using data from previous experiments. Based on the previous assumptions the mean values standard deviation and up to a 20% change in means from these previous studies dictate that 6-10 animals are to be used per group.

2.Blood pressure, respiratory parameters and temperature were measured. Regional cerebral blood flow at 24 hours after MCAO were recorded using laser-Doppler flowmetry monitor as prescribed[1,2].

**References**

# 1.Tsuchidate R, He QP, Smith ML, et al.Regional cerebral blood flow during and after 2 hours of middle cerebral artery occlusion in the rat.J Cereb Blood Flow Metab1997; 17:1066-1073.

# 2.Mao Y, Yang G, Zhou L.Temporary and permanent focal cerebral ischemia in the mouse: assessment of cerebral blood flow brain damage and blood-brain barrier permeability.Chin Med J (Engl) 2000; 113:361-366.

**Table 1.** Animal number (Survival/total) in each group.

| **Experimental 1** | | | | | |  |  | **Experimental 2** | | | | | |
| --- | --- | --- | --- | --- | --- | --- | --- | --- | --- | --- | --- | --- | --- |
| **Group**  **(time course)** | **n** | **Group**  **(IF)** | **n** | **Group**  **(FC)** | **n** |  |  | **Group**  **(MCAO24h)**  **(outcome/WB)** | **n** | **Group**  **(MCAO72h)**  **(outcome)** | **n** | **Group**  **(Elisa)** | **n** |
| **sham** | **6/6** | **sham** | **4/4** | **sham** | **6/6** |  |  | **sham** | **12/12** |  |  | **sham** | **6/6** |
| **MCAO**  **6h** | **6/6** | **MCAO 24h** | **4/5** | **MCAO 24h** | **6/6** |  |  | **MCAO**  **+vehicle** | **12/14** | **MCAO**  **+vehicle** | **6/7** | **MCAO**  **+vehicle** | **6/7** |
| **MCAO 12h** | **6/7** |  |  |  |  |  |  | **MCAO**  **+INT777(l)** | **6/7** | **MCAO**  **+INT777(m)** | **6/6** | **MCAO**  **+INT777(m)** | **6/6** |
| **MCAO 24h** | **6/7** |  |  |  |  |  |  | **MCAO**  **+INT777(m)** | **12/14** |  |  |  |  |
| **MCAO 72h** | **6/7** |  |  |  |  |  |  | **MCAO**  **+INT777(h)** | **6/7** |  |  |  |  |
|  |  |  |  |  |  |  |  |  |  |  |  |  |  |

**FC: flow cytometry ;IF:immunoﬂuorescence; IP: immunoprecipitation; WB: western blots; 10 excluded animals were not added in this table**

**Table 1.** Animal number (Survival/total) in each group. (continued)

| **Experimental 2** |  |  | **Experimental 3** | |  | **Experimental 4** | | | | | | | | | | | | | |  |  |
| --- | --- | --- | --- | --- | --- | --- | --- | --- | --- | --- | --- | --- | --- | --- | --- | --- | --- | --- | --- | --- | --- |
| **Group (long term)**  **(water maze)** |  |  | **Group**  **(co-ip)** | **n** |  | **Group**  **(outcome/ WB)** | | **n** | | **Group**  **(outcome/ WB)** | | **n** | | **Group**  **(outcome/WB)** | | **n** | |  |  |  |  |
| **sham** | **10/10** |  | **sham** | **6/6** |  | **Sham** | | **12/12** | | **sham** | | **12/12** | | **sham** | | **12/12** | |  |  |  |  |
| **MCAO**  **+vehicle** | **10/14** |  | **MCAO**  **+vehicle** | **6/7** |  | **MCAO**  **+vehicle** | | **12/14** | | **MCAO**  **+vehicle** | | **12/14** | | **MCAO**  **+vehicle** | | **12/14** | |  |  |  |  |
| **MCAO**  **+INT777(m)** | **10/14** |  | **MCAO**  **+INT777(m)** | **6/7** |  | **MCAO+**  **Scr siRNA** | | **12/14** | | **MCAO+**  **Pellino3 siRNA** | | **12/13** | | **MCAO**  **+INT777(m)** | | **12/14** | |  |  |  |  |
|  |  |  |  |  |  | **MCAO+**  **TGR5 siRNA** | | **12/14** | | **MCAO+**  **Z-IETD** | | **12/14** | | **MCAO+INT777(m)**  **+Scr siRNA** | | **12/14** | |  |  |  |  |
|  |  |  |  |  |  |  | |  | |  | |  | | **MCAO+INT777(m)**  **+TGR5 siRNA** | | **12/14** | |  |  |  |  |
|  |  |  |  |  |  |  | |  | |  | |  | | **MCAO+INT777(m)**  **+Pellino3 siRNA** | | **12/14** | |  |  |  |  |
|  |  |  |  |  |  |  |  | |  | |  | |  | |  | |  | |  | |  |

**FC: flow cytometry ;IF:immunoﬂuorescence; IP: immunoprecipitation; WB: western blots; 10excluded animals were not added in this table**

**Table 2**. Physiological parameters

| **T** | Sham | MCAO+Vehicle | MCAO+INT7777 | | |
| --- | --- | --- | --- | --- | --- |
|  |  |  | 0.16mg/kg | 0.48mg/kg | 1.44mg/kg |
| Before ischemia |  |  |  |  |  |
| MABP(mmHg） | 98±6 | 99±5 | 100±6 | 101±7 | 100±5 |
| Arterial pH | 7.28±0.03 | 7.3±0.04 | 7.31±0.04 | 7.26±0.03 | 7.24±0.03 |
| PaO_2_ (mm Hg) | 98±6.3 | 99±5.4 | 98±7.1 | 100±4.3 | 101±6.7 |
| PaCO_2_ (mm Hg) | 54.3±4.9 | 54.1±5.3 | 55.2±6.4 | 53.8±4.7 | 55.3±5.7 |
| Temperature (°C) | 37±0.18 | 36.9±0.24 | 37±0.15 | 36.9±0.31 | 37.1±0.18 |
| After ischemia |  |  |  |  |  |
| MABP | 99±6 | 98±5 | 100±6 | 99±6 | 100±4 |
| Arterial pH | 7.26±0.04 | 7.25±0.03 | 7.25±0.04 | 7.25±0.05 | 7.26±0.04 |
| PaO_2_ (mm Hg) | 99±6.4 | 101±4.6 | 102±5.6 | 101±6.4 | 99±4.8 |
| PaCO_2_ (mm Hg) | 54.5±6.6 | 57.6±7.1 | 57.6±7.6 | 56.3±5.8 | 56.3±5.4 |
| Temperature (°C) | 37.1±0.15 | 36.9±0.38 | 37.2±0.22 | 36.9±0.35 | 36.9±0.33 |

**Supplemental Figure Legends:**

**Fig.1**Experimental design and animal group classification. IF, immunoﬂuorescence; icv, intracerebral ventricular; MCAO, middle cerebral artery occlusion; Scr siRNA, Scramble small interfering RNA; WB, western blot; Co-IP, Co-immunoprecipitation.

**Fig.2** Expression of Pellino3 after middle cerebral artery occlusion (MCAO). Double immunoﬂuorescence staining for Pellino3 (red) in microglia (Iba-1, red), neuron (red) in the penumbra following MCAO. n=4 per group.

**Fig.3** **a** Triple-fluorescence staining showed that TGR5 and pellino3 colocalized in the microglia after middle cerebral artery occlusion (MCAO), n=4 per group. Scale bar, 10 μm. **b,c** Effect of Pellino3 siRNA and caspase-8 inhibitor on NLRP3 expression after MCAO, Western blot analysis and relative density. n=6 per group. **P*<0.05 vs sham, ^#^*P*<0.05 vs MCAO+ vehicle. Scr siRNA, Scramble small interfering RNA. **d** Effect of Pellino3 siRNA and caspase-8 inhibitor on infarct volume and neurobehavioral deficits. n=6 for each group. **P*<0.05 vs sham, ^#^*P*<0.05 vs MCAO + vehicle. Bars represent mean±SD.
